# The MGF360-16R ORF of African Swine Fever Virus Strain Georgia Encodes for a Nonessential Gene That Interacts with Host Proteins SERTAD3 and SDCBP

**DOI:** 10.3390/v12010060

**Published:** 2020-01-03

**Authors:** Elizabeth Ramírez-Medina, Elizabeth A. Vuono, Lauro Velazquez-Salinas, Ediane Silva, Ayushi Rai, Sarah Pruitt, Keith A. Berggren, James Zhu, Manuel V. Borca, Douglas P. Gladue

**Affiliations:** 1 Agricultural Research Service, Plum Island Animal Disease Center, Greenport, NY 11944, USA; elizabeth.ramirez@usda.gov (E.R.-M.); elizabeth.vuono@usda.gov (E.A.V.); Lauro.velazquez@usda.gov (L.V.-S.); ediane.silva@usda.gov (E.S.); ayushi.rai@usda.gov (A.R.); sarah.pruitt@usda.gov (S.P.); keith.a.berggren@gmail.com (K.A.B.); James.Zhu@usda.gov (J.Z.); 2 Department of Pathobiology, University of Connecticut, Storrs, CT 06268, USA; 3 Department of Pathobiology and Population Medicine, Mississippi State University, P.O. Box 6100, Starkville, MS 39762, USA; 4 College of Veterinary Medicine, Kansas State University, Manhattan, KS 66506, USA; 5 Oak Ridge Institute for Science and Education, Oak Ridge, TN 37830, USA

**Keywords:** ASFV, African swine fever virus, ASF, host–viral protein interactions, SDCP1, SERTAD3

## Abstract

African swine fever virus (ASFV) causes a contagious and frequently lethal disease of pigs with significant economic consequences to the swine industry. The ASFV genome encodes for more than 160 genes, but only a few of them have been studied in detail. Here we report the characterization of open reading frame (ORF) MGF360-16R. Kinetic studies of virus RNA transcription demonstrated that the MGF360-16R gene is transcribed as a late virus protein. Analysis of host–protein interactions for the MGF360-16R gene using a yeast two-hybrid screen identified SERTA domain containing 3 (SERTAD3) and syndecan-binding protein (SDCBP) as host protein binding partners. SERTAD3 and SDCBP are both involved in nuclear transcription and SDCBP has been shown to be involved in virus traffic inside the host cell. Interaction between MGF360-16R and SERTAD3 and SDCBP host proteins was confirmed in eukaryotic cells transfected with plasmids expressing MGF360-16R and SERTAD3 or SDCBP fused to fluorescent tags. A recombinant ASFV lacking the MGF360-16R gene (ASFV-G-ΔMGF360-16R) was developed from the highly virulent field isolate Georgia2007 (ASFV-G) and was used to show that MGF360-16R is a nonessential gene. ASFV-G-ΔMGF360-16R had a similar replication ability in primary swine macrophage cell cultures when compared to its parental virus ASFV-G. Experimental infection of domestic pigs showed that ASFV-G-ΔMGF360-16R is as virulent as the parental virus ASFV-G.

## 1. Introduction

African swine fever virus (ASFV) causes a contagious and frequently lethal viral disease of swine, African swine fever (ASF). ASFV is a double-stranded DNA virus that contains approximately 180–190 kilobase pairs [1]. ASF causes a disease with various outcomes from highly lethal to subclinical depending both on the viral strain and on the host species that is infected [2]. In domestic pigs, virulent strains are fatal with symptoms of fever, general hemorrhages, ataxia, and severe depression.

Currently, there is no commercial vaccine available for ASF. Outbreaks of ASF are controlled by culling of animals in infected farms. ASF has been historically endemic in several sub-Saharan African countries and has been endemic in Sardinia (Italy). Recent outbreaks outside this area started in the Caucasus, first affecting Georgia in 2007, followed by Armenia, Azerbaijan, and Russia. More recently, outbreaks have been found in several countries in East Europe, threatening to disseminate into other West European countries [3]. In 2018 the ASFV outbreak was introduced into China and has quickly spread throughout Southeast Asia.

The ASFV genome consists of at least 160 open reading frames (ORFs) with at least 100 proteins detected by mass spectrometry in viral particles [4,5,6,7,8,9]. However, the function of most of these ORFs has not been experimentally tested and is often attributed solely based on sequence homology prediction to cellular and other related viral proteins. The role of the specific ASFV genes that contribute to viral virulence or for virus host range to specific host species still remains unknown [2].

In this study, we examined the role of a previously uncharacterized ASFV protein, encoded by ORF multigene family 360-16R (MGF360-16R), which is a highly conserved protein among ASFV isolates. ASFV-G has open reading frames for 15 members of the MGF360 gene family scattered on both ends of the genome, with 13 of them at the beginning in the left terminal repeat region (MGF360-1L, 2L, 3L, 4L, 6L, 8L, 9L, 10L, 11L, 12L, 13L, 14L, 15L) and 2 at the end of the genome in the right terminal repeat region (MGF360-16R and 21R). Previous studies have shown that some of the MGF360 genes on the left arm of the genome can be partially deleted along with other viral genes during stable cell adaptation, leading to decreased virulence in swine [10]. We have previously shown that deleting six MGF genes located in the left variable region, three from the MGF360 family of genes and three from the MGF505 family of genes, resulted in full attenuation in swine and, more importantly, protection against homologous wild-type (WT) challenge [11]. We have also previously shown that deletion of both MGF360-13L and MGF360-14L in ASFV-G led to a phenotype indistinguishable from that of the parental virus [12]. Another study showed that using a recombinant virus with a large deletion of the MGF360 family along with the MGF505 family did not induce circulating interferon when compared to the parental WT virus, and that cell culture interferon could inhibit nonvirulent ASFV with a deletion of similar MGF genes [13]. In *Ornithodoros porcinus* ticks, it has been shown that other MGF360 genes are required for replication and generalization of infection [14]. However, to date, no one has investigated the role of any individual MGF genes, and MGF360-16R has not been part of any larger deletions though virus adaptation or in any recombinant virus study. The function of individual MGF genes and the interaction of their translated protein products with host proteins remains unknown. 

In this study, it was determined that MGF360-16R is a nonessential gene that is transiently expressed as a late virus protein. Recombinant ASFV with a deletion in the MGF360-16R gene (ASFV-G-ΔMGF360-16R) was shown to have a similar ability to replicate in primary swine macrophage cell cultures when compared to its parental virus ASFV-G. It is also shown that MGF360-16R specifically interacts with SERTA domain containing 3 (SERTAD3) and syndecan-binding protein (SDCBP), indicating a potential role for MGF360-16R in modulating transcriptional activity of the host cell. Nevertheless, infection of domestic pigs showed that ASFV-G-ΔMGF360-16R produces a disease similar to the virulent parental virus ASFV-G.

## 2. Materials and Methods

### 2.1. Cell Cultures, Viruses, and Growth Curves

Primary swine macrophage cell cultures were prepared from defibrinated swine blood as previously described [15]. Briefly, heparin-treated swine blood was incubated at 37 °C for 1 h to allow sedimentation of the erythrocyte fraction. Mononuclear leukocytes were separated by flotation over a Ficoll-Paque (Pharmacia, Piscataway, NJ, USA) density gradient (specific gravity, 1.079). The monocyte/macrophage cell fraction was cultured in plastic Primaria (Falcon; Becton Dickinson Labware, Franklin Lakes, NJ, USA) tissue culture flasks containing macrophage medium, composed of RPMI 1640 Medium (Life Technologies, Grand Island, NY, USA), 30% in-house cultured L929 supernatant, and 20% fetal bovine serum (HI-FBS, Thermo Scientific, Waltham, MA, USA), for 48 h at 37 °C under 5% CO_2_. Adherent cells were detached from the plastic by using 10 mM EDTA in phosphate-buffered saline (PBS) and were then reseeded into Primaria T25 in 6- or 96-well dishes at a density of 5 × 10^6^ cells per ml for use in assays 24 h later.

ASFV Georgia (ASFV-G) was a field isolate kindly provided by Dr. Nino Vepkhvadze, from the Laboratory of the Ministry of Agriculture (LMA) in Tbilisi, Republic of Georgia [10]. 

Comparative growth curves between ASFV-G-ΔMGF360-16R and parental ASFV-G were performed in primary swine macrophage cell cultures. Preformed monolayers were prepared in 24-well plates and infected at a MOI of 0.1 (based on the HAD_50_ value previously determined in primary swine macrophage cell cultures). After 1 h of adsorption at 37 °C under 5% CO_2_, the inoculum was removed and the cells were rinsed two times with PBS. The monolayers were then rinsed with macrophage medium and incubated for 2, 24, 48, 72, and 96 h at 37 °C under 5% CO_2_. At appropriate times post infection, the cells were frozen at ≤−70 °C and the thawed lysates were used to determine titers by HAD_50_/mL in primary swine macrophage cell cultures. All samples were run simultaneously to avoid interassay variability.

Virus titration was performed on primary swine macrophage cell cultures in 96-well plates. Virus dilutions and cultures were performed using macrophage medium. Presence of virus was assessed by hemadsorption (HA) and virus titers were calculated as previously described [16].

### 2.2. Microarray Analysis

The microarray data of ASFV open reading frames were obtained from the dataset deposited in the NCBI database [17]. In brief, total RNA was extracted from primary swine macrophage cell cultures infected with ASFV Georgia strain or mock infected at 3, 6, 9, 12, 15, and 18 h post infection (hpi). A custom-designed porcine microarray manufactured by Agilent Technologies (Chicopee, MA, USA) was used for this study. Both infected and mock-infected RNA samples were labeled with Cy3 and Cy5 using an Agilent low-input RNA labeling kit (Agilent Technologies, Santa Clara, CA, USA). Cy5-labeled infected or mock-infected samples were co-hybridized with Cy3-labeled mock-infected or infected samples in one array, respectively, for each time point using a dye-swap design. The entire procedure of microarray analysis was conducted using the protocols, reagents, and equipment provided or recommended by Agilent Technologies. Array slides were scanned using a GenePix 4000B scanner (Molecular Devises, San Jose, CA, USA) with GenePix Pro 6.0 software at 5 μm resolution. Background signal correction and data normalization of the microarray signals and statistical analysis were performed using the LIMMA package. The signal intensities of ASFV open reading frame RNA were averaged from both Cy3 and Cy5 channels.

### 2.3. Construction of the Recombinant Viruses

**Figure 1:**
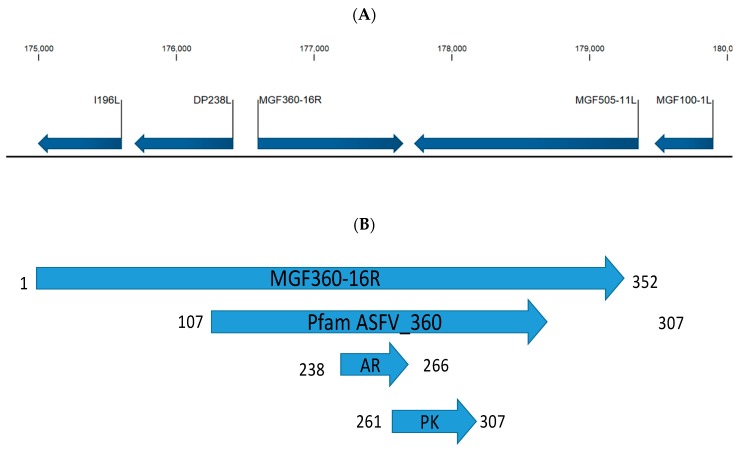
MGF360-16R open reading frame (ORF) description. (**A**) Schematic representation of the MGF360-16R ORF region in the African swine fever virus (ASFV)-G genome, showing adjacent open reading frames. (**B**) Schematic representation of the predicted domains in the MGF360-16R protein. Amino acid positions of the domains are on the ends of the protein domain. The domains include the pfam MGF360 domain, the ankyrin repeat domain (AR), and the protein-kinase-like domain (PK).

Recombinant ASFV-G-ΔMGF360-16R was generated by homologous recombination between the parental ASFV genome and a recombination transfer vector following previously described procedures [15,18]. The recombinant transfer vector (p72mCherryΔMGF360-16R) contained flanking genomic regions—the left arm is located between genomic positions 175589 and 176589 and the right arm is located between genomic positions 177312 and 178312—and a reporter gene cassette containing the mCherry fluorescent protein (mCherry) gene under the control of the ASFV p72 late gene promoter [12]. The recombinant transfer vector was obtained by DNA synthesis (Epoch Life Sciences, Sugar Land, TX, USA). This construction created a 722-nucleotide deletion between nucleotide positions 176,590 and 177,311 inclusive, deleting most of the ORF sequence for MGF360-16R with the coding region for the last 111 amino acids of the C-terminus remaining, but without a promoter or start codon, making the transcription of this product unlikely (Figure 1). Macrophage cell cultures were infected with ASFV-G and transfected with p72mCherryΔMGF360-16R. Recombinant ASFV-G-ΔMGF360-16R was purified to homogeneity by successive rounds of limiting dilution purification.

### 2.4. Library Screening

The GAL4-based yeast two-hybrid system was used for this study [19,20]. The “bait” protein, ASFV Georgia MGF360-16R (nucleotide residues 176,590–177,519 of the ASFV Georgia genome), was expressed with an N-terminus fusion to the GAL4 binding domain [21]. As “prey”, a swine macrophage cDNA library that was constructed using swine monocytes/macrophage cDNA (as previously described [22]) containing proteins fused to the GAL4 activation domain (AD) was used. Screening was done as previously described [23].

### 2.5. Colocalization Studies

GFP-MGF360-16R was inserted into pTagGFP-N1 where GFP-MGF360-16R is under the control of the CMV promoter, and RFP-SERTAD3 and RFP-SDCBP were cloned into pTagRFP-N; both plasmids were constructed by Epoch Life Sciences. Cell fixation and imaging was done as previously described [24].

### 2.6. Next-Generation Sequencing (NGS) of ASFV Genome

ASFV DNA was extracted from infected cells and quantified as described earlier. Full-length sequencing of the virus genome was performed as described previously [25] using an Illumina NextSeq500 sequencer.

### 2.7. Animal Experiments

ASFV-G-ΔMGF360-16R was assessed for its virulence phenotype relative to the parental ASFV-G virus using 80–90 pound commercial breed swine. Five pigs were inoculated intramuscularly (IM) with 10^2^ HAD_50_ of ASFV-G-ΔMGF360-16R and compared with a group of pigs inoculated with similar doses of ASFV-G. Clinical signs (anorexia, depression, fever, purple skin discoloration, staggering gait, diarrhea, and cough) and changes in body temperature were recorded daily throughout the experiment.

### 2.8. Ethics Statement

Animal experiments were performed under biosafety level 3AG conditions in the animal facilities at Plum Island Animal Disease Center (PIADC). All experimental procedures were carried out in compliance with the Animal Welfare Act (AWA), the 2011 Guide for Care and Use of Laboratory Animals, the 2002 PHS Policy for the Humane Care and Use of Laboratory Animals, and U.S. Government Principles for Utilization and Care of Vertebrate Animals Used in Testing, Research and Training (IRAC 1985), as well as specific animal protocols reviewed and approved by the PIADC Institutional Animal Care and Use Committee of the U.S. Departments of Agriculture and Homeland Security (protocol number 225.04-16-R, 09-07-16).

## 3. Results

### 3.1. MGF360-16R Gene Is Conserved across Different ASFV Isolates

**Figure 2:**
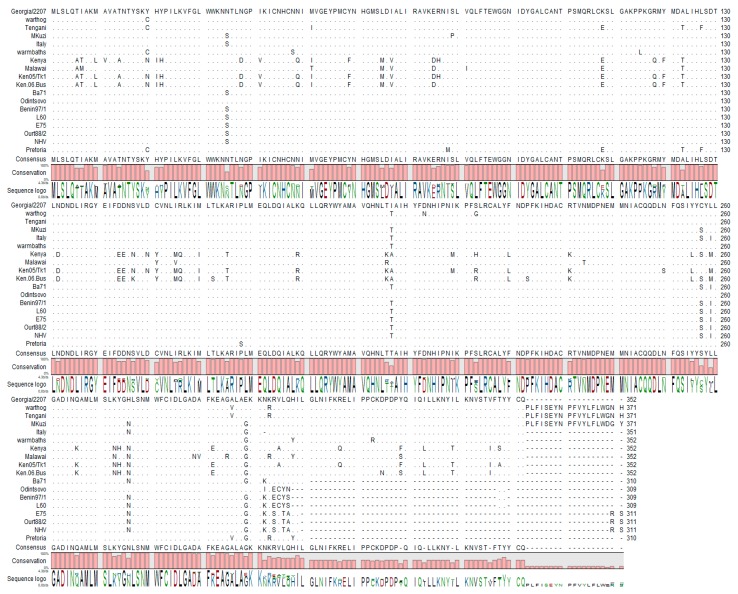
Multiple sequence alignment of the indicated ASFV isolates of viral protein MGF360-16R. Matching residues are represented as dots. The degree of conservation is presented below the protein sequence, and the conserved residue is presented on the bottom, indicating the degree of conservation for particular amino acids in the protein sequence.

ASFV ORF MGF360-16R encodes for a 352-amino-acid protein; it is encoded on the forward strand in the right variable region of the ASFV-G genome, nucleotide positions 176,590 to 177,647 (Figure 1A), and is located between ORFs DP238L and MGF505-11L. At the amino acid level the MGF360-16R protein contains three domains predicted by SMART [26]—amino acids 107–307 encode for the Pfam ASFV360 domain which characterizes this protein as a member of the MGF360 family [27]. There is an ankyrin repeat domain between residues 238 and 266; ankyrin domains are one of the most abundant protein–protein interaction domains in nature. Between residues 261 and 307 there is a domain that resembles a putative protein-kinase-like domain (Figure 1B). The degree of MGF360-16R conservation among ASFV isolates was examined by multiple alignment using CLC Genomics Workbench 12 (https://www.qiagenbioinformatics.com/) (Figure 2). The ASFV MGF360-16R gene sequences were derived from all sequenced isolates of ASFV representing African, European, and Caribbean isolates from both pig and tick sources. In most isolates, including ASFV-G, MGF360-16R has a predicted protein of 309–371 amino acid residues in length. Recently it was shown that there was an adenosine repeat that resulted in there being eight, not seven, adenosines with an insertion at position 177,493; this results in the fusion of the annotated MGF360-16R ORF and the DP63R ORF in the initial annotation of Georgia 2007/1 [8]. We confirmed that in our ASFV-G there are eight adenosines. It is possible that the differences in length observed in the MGF360-16R ORFs in some of the different isolates are due to potential sequences having this series of repeating adenosines misread by the sequencing technology used at the time the isolate was sequenced. Regardless, there is a high degree of homology between all isolates in the region of the first 309 amino acids and a high degree of homology between amino acids 309 and 352 for isolates that were sequenced to show an MGF360-16R gene of this size. Regardless of size, all isolates have 77% invariant residues when compared with all other isolates (Figure 2).

### 3.2. MGF360-16R Is Transcribed as a Late Viral Gene

**Figure 3:**
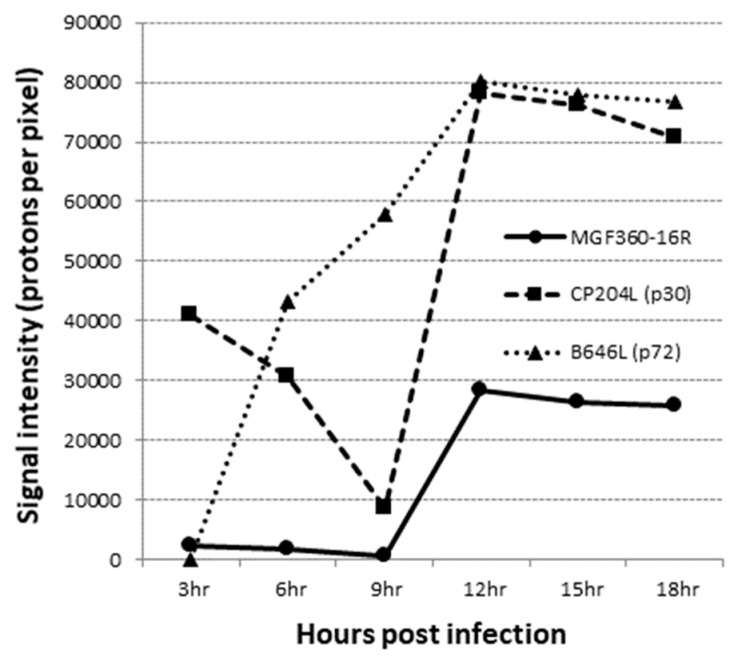
Time course of MGF360-16R gene transcriptional activity. Averaged microarray signal intensities (photons per pixel) of ASFV MGF360-16R, CP204L, and B646L open reading frame RNA prepared from ex vivo pig macrophages infected with ASFV at 3, 6, 9, 12, 15, and 18 h post infection.

To determine whether the MGF360-16R gene is actually transcribed during the infectious cycle, a time course experiment was performed to analyze the kinetics of RNA transcription in primary swine macrophages infected with ASFV strain Georgia. Swine macrophage cultures were infected (MOI = 10) with ASFV-G and cell lysate samples were taken at 3, 6, 9,12, 15, and 18 h post infection (hpi), approximately completing one virus replication cycle. The presence of MGF360-16R RNA was detected by DNA microarray analysis, as described in Section 2.2. Transcription of MGF360-16R was reliably detected at all time points, with signal-to-noise ratios of 18 or larger (SNR of 3 is the threshold of reliable microarray detection); expression gradually decreased from 3 to 9 hpi and then peaked at 12 hpi, followed by a decrease from 12 to 18 hpi, like the trend observed during the period of 3 to 9 hpi (Figure 3). The pattern of expression of the well-characterized ASFV early protein p30 (CP204L) and the late protein p72 (B646L) was used as a control for protein expression (expression of a housekeeping gene, α-tubulin, was used as a loading control). Therefore, the ASFV MGF360-16R gene encodes for a protein that is highly expressed late in the virus replication cycle.

### 3.3. MGF360-16R Specifically Binds Swine Host Proteins

**Figure 4:**
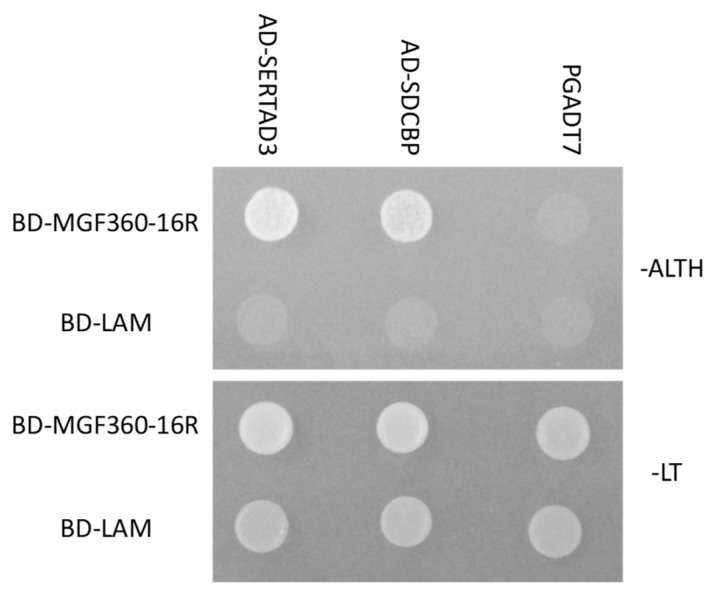
Protein–protein interaction of ASFV MGF360-16R with swine SERTA domain containing 3 (SERTAD3) and syndecan-binding protein (SDCBP) in the yeast two-hybrid system. Spots of strains, 10 μL, expressing the indicated constructs containing 2 × 10^6^ yeast cells were spotted on selective media (top panel) for protein–protein interaction in the yeast two-hybrid system, SD–Ade/His/Leu/Trp plates (-ALTH), and nonselective media (bottom panel) SD–Leu/Trp (-LT) for plasmid maintenance only.

In order to understand the possible role of MGF360-16R in virus replication or virulence we utilized a yeast two-hybrid system using a custom swine macrophage protein library to identify swine host proteins possibly interacting with MGF360-16R. An N-terminal fusion of the GAL4 DNA binding domain [21] with the ASFV MGF360-16R protein was utilized as “bait” to screen a swine primary macrophage cDNA library expressed as N-terminal fusions of the GAL4 activation domain (AD). We screened more than 1 × 10^7^ independent yeast colonies, from a swine primary macrophage cDNA library containing 3 × 10^6^ independent clones. These colonies were selected for growth on Leu/-Trp/-His/-Ade medium. Plasmids were isolated from positive colonies and sequenced. In-frame proteins were retested for specificity to the MGF360-16R protein. As a negative control, proteins were tested in their ability to bind to the human Lamin C (Lam-BD) protein. SERTA domain-containing protein 3 (SERTAD3) and syndecan-binding protein (SDCBP) were among the swine host proteins identified as specific binding partners for the ASFV MGF360-16R protein (Figure 4).

**Figure 5:**
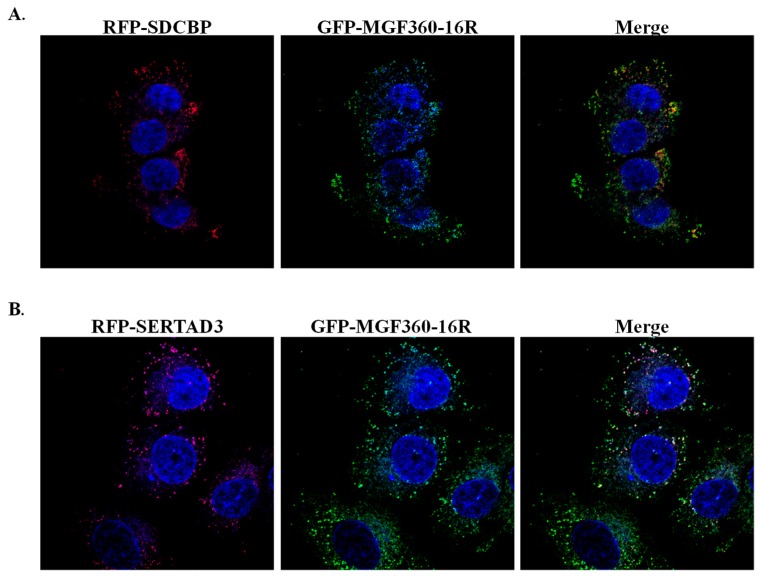
Protein–protein interaction of ASFV MGF360-16R protein with swine SERTAD3 and SDCBP in eukaryotic cells. Colocalization of MGF360-16R and either (**A**) SERTAD3 or (**B**) SDCBP in swine kidney (SK6) cells imaged at 1000×. GFP-MGF360-16R-GFP and RFP-SERTAD3 or RFP-SDCBP constructs were co-transfected in SK6 cell cultures as indicated. Yellow color in the merged panel indicates the presence of co-localization between RFP and GFP.

To further confirm the interaction of MGF360-16R and the identified host proteins in swine cells, MGF360-16R was tagged on the N-terminus with GFP (GFP-MGF360-16R) and SERTAD3 and SDCBP were tagged with RFP on the N-terminus (RFP-SERTAD3 and RFP-SDCBP). MGF360-16R-GFP and either RFP-SERTAD3 or RFP-SDCBP plasmids were co-transfected into swine kidney cells (SK6) and observed for colocalization. In both cases, colocalization was observed in cells with both plasmids expressed where MGF360-16R and SERTAD3 or SDCBP both make numerous punctuated overlapping structures throughout the cell cytoplasm, confirming that MGF360-16R and both SERTAD3 and SDCBP co-localize in swine cells (Figure 5).

### 3.4. Development of the ASFV-G-ΔMGF360-16R Deletion Mutant

**Figure 6:**
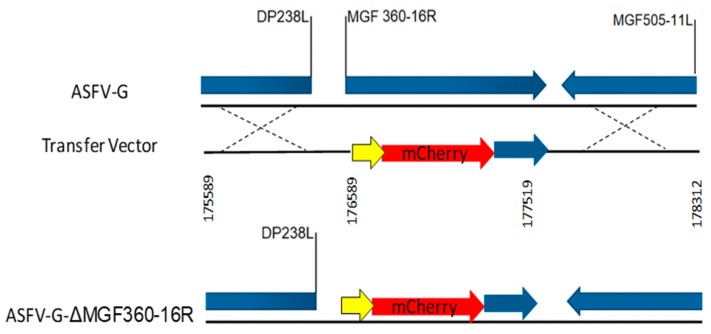
Schematic for the development of ASFV-G-ΔMGF360-16R. The transfer vector contains p72 promoter, as indicated with the yellow arrow, and an mCherry cassette, shown in red; the nucleotide positions of the flanking left and right arms are indicated and were designed to have flanking ends to both sides of the deletion/insertion cassette, indicated by the dashed lines. The resulting ASFV-G-ΔMGF360-16R virus with the cassette inserted is shown on the bottom.

To determine the role of MGF360-16R during ASFV infection in cell cultures and virulence in swine, a recombinant virus lacking the MGF360-16R gene was designed. Deletion of MGF360-16R was achieved by replacing the 1–241 amino acid residues of the ORF with the p72mCherry cassette following standard methodologies based on homologous recombination. The designed recombinant virus, ASFV-G-ΔMGF360-16R, was constructed from the highly pathogenic ASFV Georgia 2007 isolate (ASFV-G). A 722 bp region was deleted (between nucleotide positions 175590 and 177311) from ASFV-G virus and replaced with a 1294 bp cassette containing the p72mCherry (see Section 2.3). The resulting recombinant virus harbors a deletion in the MGF360-16R ORF (Figure 6) leaving the C-terminal 333 bp as to not disrupt the promotor or coding region of DP63R. The recombinant virus was obtained after successive limiting dilution purification events on monolayers of primary swine macrophage cell cultures. The virus population obtained from the last round of purification was amplified in primary swine macrophage cell cultures to obtain a virus stock.

To evaluate the accuracy of the genetic modification and the integrity of the genome of the recombinant virus, full genome sequences of ASFV-G-ΔMGF360-16R and parental ASFV-G were obtained by NGS on an Illumina NextSeq^®^ 500 and compared. The full-length genome comparison between ASFV-G-ΔMGF360-16R and parental ASFV-G revealed a deletion of 722 nucleotides corresponding with the introduced modification. Additionally, the consensus sequence of the ASFV-G-ΔMGF360-16R genome showed an insertion of 1294 nucleotides corresponding to the p72-mCherry cassette sequence. Besides the insertion cassette containing the reporter gene, no additional significant differences were observed between the ASFV-G-ΔMGF360-16R and ASFV-G genomes, confirming that the ASFV-G-ΔMGF360-16R virus did not accumulate mutations during the process of homologous recombination and plaque purification. In addition, NGS confirmed the absence of any residual parental ASFV-G genome as a contaminant of the ASFV-G-ΔMGF360-16R stock.

### 3.5. Replication of ASFV-G-ΔMGF360-16R in Primary Swine Macrophages

**Figure 7:**
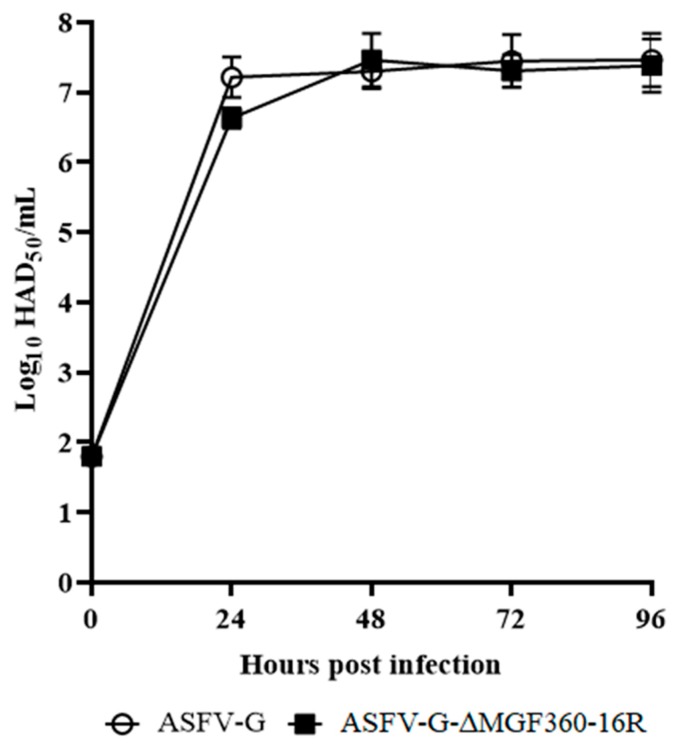
In vitro growth kinetics in primary swine macrophage cell cultures infected (MOI = 0.01) with either ASFV-G-MGF360-16R or parental ASFV-G virus. Samples were taken from three independent experiments at the indicated time points and titrated. Data represent means and standard deviations. The sensitivity using this methodology for detecting virus was >log_10_ 1.8 50% hemadsorbing doses per ml (HAD_50_/mL).

To evaluate the role of the MGF360-16R gene in virus replication, the in vitro growth characteristics of ASFV-G-ΔMGF360-16R were assessed in primary swine macrophage cell cultures, the primary cell targeted by ASFV during infection in swine, and compared to parental ASFV-G via a multistep growth curve. Cell cultures were infected with these viruses at a MOI of 0.01 and samples were collected at 2, 24, 48, 72, and 96 hpi. ASFV-G-ΔMGF360-16R displayed almost identical growth kinetics when compared to the parental ASFV-G virus (Figure 7). Therefore, deletion of MGF360-16R genes does not significantly affect the ability of the virus to replicate in primary swine macrophage cultures. 

### 3.6. Assessment of ASFV MGF360-16R Virulence in Swine

**Figure 8:**
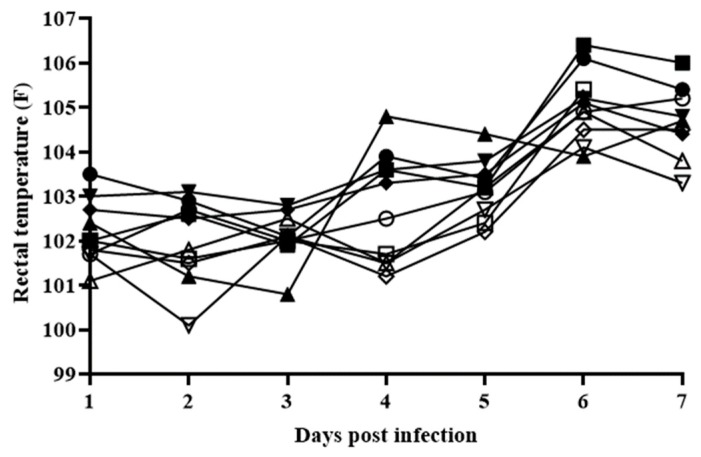
Kinetics of body temperature values in pigs intramuscularly (IM) inoculated with 10^2^ HAD_50_ of either ASFV-G-ΔMGF360-16R (filled symbols), or ASFV-G (empty symbols). Each curve represents individual animal values in each of the groups.

In order to evaluate the effect of the deletion of the MGF360-16R gene on ASFV-G virulence, a group of five 80–90 pound pigs was inoculated intramuscularly (IM) with 10^2^ HAD_50_ per animal, while a control group received IM 10^2^ HAD_50_ of ASFV-G. As expected, animals in the group infected with ASFV-G exhibited increased body temperature (>104 °F) by Day 6 pi followed by the appearance of clinical signs associated with the disease, including anorexia, depression, purple skin discoloration, staggering gait, and diarrhea (Table 1 and Figure 8). 

Signs of the disease quickly worsened and animals were euthanized in extremis by Day 7–8 pi. Interestingly, animals receiving ASFV-G-ΔMGF360-16R presented a clinical disease indistinguishable from that presented by those inoculated with ASFV-G. Both time of presentation and severity of the clinical signs related to the disease resembled those present in animals inoculated with the parental virus. Therefore, deletion of the ASFV-G-ΔMGF360-16R gene does not alter the virulence phenotype of the highly virulent ASFV-G isolate.

**Figure 9:**
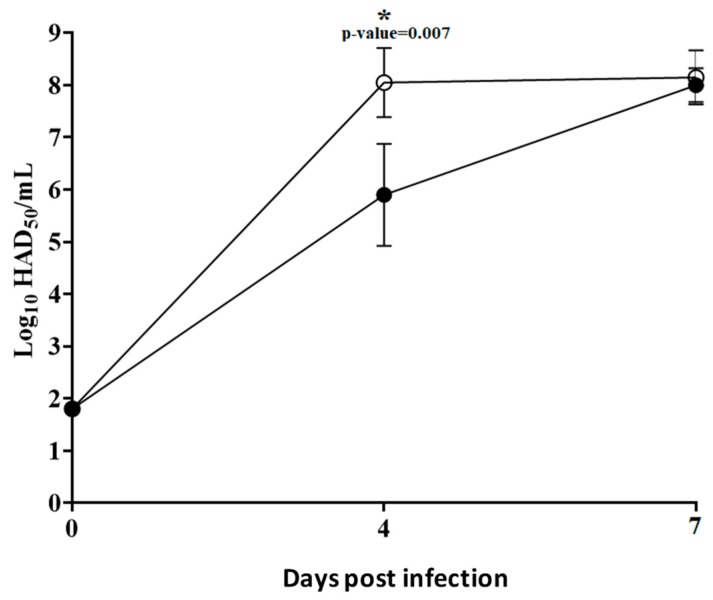
Viremia titers detected in pigs IM inoculated with 10^2^ HAD_50_ of either ASFV-G-ΔMGF360-16R (filled symbols), or ASFV-G (empty symbols). Each curve represents the average of animal values in each of the groups. Sensitivity of virus detection: >log_10_ 1.8 TCID_50_/mL.

Analysis of viremia in animals infected with ASFV-G presented expected high titers (10^7^–10^8.5^ HAD_50_/mL) on Day 4 pi, remaining high until Day 7 pi when all animals were euthanized. ASFV-G-ΔMGF360-16R infected animals had viremias with values ranging from 10^4^ to 10^6.5^ HAD_50_/mL by Day 4 pi, reaching titers similar to those of animals infected with ASFV-G by Day 7 pi, which was the last sampling time before animals were humanely euthanized (Figure 9). Therefore, ASFV-G-ΔMGF360-16R virulence, in terms of clinical presentation and virological data, produces a disease indistinguishable from that induced by its highly virulent parental virus, ASFV-G.

## 4. Discussion

The ASFV genome encodes for more than 160 proteins, but very few of them have been characterized to date. Knowing the function of ASFV proteins is important to understanding virus replication and virulence in swine, which, in turn, is important to facilitating the development of novel countermeasures to control the disease. The functional characterization of virus genes has allowed the development of potential ASFV attenuated strains that have been used as experimental vaccine candidates [11,28,29,30]. In that regard, a restricted number of virus genes have been successfully deleted in several recombinant viruses using an infectious ASFV backbone (e.g., genes as 9GL, lectin, UK, MGF, NL, CD2, Lectin) [11,15,29,30,31,32,33,34,35,36,37], with a small number of genes determined to be essential for virus replication (e.g., EP152R, p30, p54, p72) [38,39,40]. This lack of information limits the current knowledge on most ASFV proteins to only ORF functional genomics and the predicted functions of these ORFs.

In this study, we determined that MGF360-16R, a previously uncharacterized ASFV ORF, is actually transcribed at late times during the infection of swine primary macrophages. It is also shown that MGF360-16R is a nonessential gene: its deletion from the ASFV-G genome does not significantly alter virus replication in swine macrophage cultures and, importantly, is not critical for ASFV virulence in swine, as ASFV-G-ΔMGF360-16R had similar pathogenesis as the parental ASFV-G.

In the field of ASFV, there still remains a great need for further experimental characterization of viral proteins and their possible role during infection in swine. Although it has been postulated that the virus modulates the host immune response to facilitate the progress of infection (reviewed by Reis et al. 2016 [37]), the precise molecular mechanisms involved allowing ASFV to manipulate host cellular processes for its own survival are still not well understood. Very few host proteins are known to directly interact with ASFV proteins [33,40,41,42], and further work is required to determine possible virus–host interactions that may be involved in determining the pathogenesis of ASFV in swine. In this study we identified that the viral protein MGF360-16R specifically binds to host proteins, genes SERTAD3 and SDCBP, regulatory factors of cell transcription. 

SDCBP has been shown to interact with several other viral proteins, for example, the HIV protein NEF, thought to be involved in promoting viral protein entry [43], as well as the Epstein–Barr virus protein LMP1 [44]. In human papillomaviruses, SDCBP, when interacting with host CD63, has been shown to control the postendocytic trafficking of these viruses [45]. SERTAD3 has been shown to be a transcriptional coactivator, with expression elevated in cancer cell lines, but very little other information is known about the role of this protein [46], particularly during virus infections. 

Regardless of the potential importance of protein interaction with multifunctional host proteins that are involved in the process of regulating both cell transcription and potential protein trafficking in the cell, deletion of the MGF360-16R gene does not seem to drastically affect the ability of ASFV to replicate in either swine macrophage cultures or, more importantly, during infection in swine or the ability of the virus to produce disease. This suggests that the loss of MGF360-16R and the ability to bind both SDCBP and SERTAD3 is nonessential for virus virulence. In a large DNA virus such as ASFV, that produces many proteins, it is possible that redundant or overlapping functions to occur between viral proteins. This could be due to multiple viral proteins binding the same cellular proteins, or due to other viral proteins binding a different cellular protein in the same pathway. Therefore, it is possible that the pathways that are being manipulated by MGF360-16R binding to SDCBP and SERTAD3 are also manipulated by other viral proteins, allowing for the lack of an observed phenotype by deletion of MGF360-16R. 

Understanding the host–viral molecular mechanisms that occur during infection can lead to a better understanding of viral pathogenesis, which in turn can allow for the construction of better rational vaccine designs.

## Figures and Tables

**Table 1:** Swine survival and fever response following infection with ASFV MGF360-16R and parental ASFV-G.

			Fever		
Virus (10^2^ HAD_50_)	No. of Survivors/Total	Mean Time to Death (±SD)	No. of Days to Onset (±SD)	Duration No. of Days (±SD)	Maximum Daily Temp., °F (±SD)
ASFV MGF360-16R	0/5	8 (0)	5.6 (0.89)	2.4 (0.89)	105.34 (0.98)
ASFV-G	0/5	7.8 (0.45)	6 (0)	1.8 (0.45)	104.76 (0.54)
